# Probabilistic comparisons of grey water footprint models for herbicide mixtures in Brazilian sugarcane

**DOI:** 10.1007/s11356-026-38125-9

**Published:** 2026-08-12

**Authors:** Lourival Costa Paraíba

**Affiliations:** Embrapa Meio Ambiente, Rodovia SP-340, Km 127,5, Tanquinho Velho, Jaguariúna, São Paulo 13918-110 Brazil

**Keywords:** GWF, Herbicide mixture, Monte Carlo, HRAC, H-model, P-model, Cumulative risk assessment

## Abstract

The grey water footprint (GWF) quantifies freshwater pollution from pesticide use. The H-model relies on regulatory concentration limits, while the P-model uses ecotoxicological endpoints and concentration addition (CA); independent action (IA) across modes of action (MOA) remains underexplored. We present a fully re-verified probabilistic Monte Carlo comparison of CA and HRAC-based IA P-models for 17 herbicides applied to Brazilian sugarcane, built exclusively from traceable, source-cited inputs, including a soil attenuation factor recomputed from first principles and maximum acceptable concentrations compiled from five source tiers. Total grey water footprint is $2.3\times 10^{12}$ m$^3$ under both CA and IA, with HRAC group C1 dominating in 100% of Monte Carlo iterations. The P-model exceeds the H-model by a factor *R* of 7 to 20 depending on data quality. Carfentrazone’s score rises from 1.5 to 4.9 once group membership is considered, and group E2’s hazard is driven mainly by oxyfluorfen. We report this audit trail transparently, prioritising numerical traceability over a single headline figure.

**Supplementary Information:**

The online version contains supplementary material available at 10.1007/s11356-026-38125-9.

## Introduction

Freshwater scarcity is among the most pressing environmental concerns worldwide (Pfister et al. 2011). As agricultural production expands to meet demand for food, fibre, and biofuels, it puts growing pressure on water resources—quantitatively, through blue and green water, and qualitatively, through grey water. The water footprint concept, developed by Hoekstra and colleagues (Hoekstra and Chapagain 2008; Hoekstra et al. 2011), captures the volume of freshwater used directly and indirectly to produce a good or service.

Water footprints split into three components: blue water, drawn from rivers, lakes, and aquifers; green water, rainwater stored in the soil; and grey water, the volume of freshwater needed to dilute pollutants down to acceptable levels. For pesticide mixtures specifically, this last component is what the grey water footprint (GWF) sets out to capture—water pollution expressed as the dilution volume required to meet water-quality standards (Barreto et al. 2020; Hoekstra et al. 2011; Paraíba et al. 2014).

The GWF for pesticides is usually calculated with the model (Hoekstra et al. 2011) proposed, which we refer to here as the H-model:

1 $$\begin{aligned} V_{GW}^{H} = 1000\,\frac{\alpha \, A_C \, A_D}{C_{max}} \end{aligned}$$

where $V_{GW}^{H}$ (m$^3$) is the grey water volume; α is the fraction of pesticide reaching water bodies via runoff and/or leaching; $A_C$ (ha) is the cultivated area; $A_D$ (kg ha$^{-1}$) is the applied dose; and $C_{max}$ (mg L$^{-1}$) is the maximum concentration of the pesticide that regulatory agencies consider acceptable in water. Since 1 mg L$^{-1}$ is numerically equal to $10^{-3}$ kg m$^{-3}$, dividing the pesticide mass $\alpha A_C A_D$ (kg) by the numeric value of $C_{max}$ in mg L$^{-1}$ understates the true concentration by a factor of 1000; the leading factor of 1000 in Eq. 1 corrects for this, so that $V_{GW}^{H}$ comes out directly in cubic metres.

Useful as it is, the H-model has three real limitations: it ignores mixture effects, it depends on regulatory standards that simply do not exist for many pesticides, and it leaves out acute toxicity to aquatic organisms. Paraíba et al. (2014) addressed this by proposing the P-model, which estimates mixture toxicity through Concentration Addition (CA) instead. The original version applies CA across the board—a reasonable choice when every pesticide in the mixture shares the same mode of toxic action (MOA) in the target organism (Backhaus et al. 2003; Loewe 1953), but not otherwise: when MOAs differ, independent action (IA) is the more defensible model (Bliss 1939), and both European legislation and international risk-assessment guidance (ECHA 2014; EFSA 2013) call for CA within a shared MOA group and IA across groups. For herbicides specifically, the Herbicide Resistance Action Committee (HRAC) classification gives us that grouping.

Sugarcane cultivation in Brazil occupies approximately $8.4\times 10^{6}$ ha (CONAB 2012) and uses a wide variety of herbicides belonging to different HRAC groups. Sugarcane is a particularly suitable case study for a mixture-based grey water footprint framework for three reasons. First, herbicide-use intensity is high relative to most other Brazilian row crops: weed competition during the slow-establishing early growth stage can reduce yield by over 30%, so growers routinely apply sequential, multi-mode-of-action herbicide programmes (often combining a pre-emergence residual herbicide from one HRAC group with a post-emergence contact herbicide from another) rather than a single active ingredient, making mixture toxicity—not single-compound toxicity—the practically relevant exposure scenario. Second, the crop’s environmental vulnerability is significant: sugarcane is concentrated in São Paulo and the Center-South region, an area with extensive surface-water and shallow-groundwater connectivity (including recharge zones of the Guarani Aquifer System), where herbicide runoff and leaching directly threaten drinking-water and aquatic-ecosystem quality. Third, sugarcane’s economic and policy importance in Brazil—as the basis of both sugar and ethanol production, the latter a renewable-fuel policy priority—means that regulatory or certification decisions affecting herbicide use in this crop have an outsized national impact, making a transparent, mixture-aware risk-ranking tool directly actionable for both growers and regulators. A recent field study by Barreto et al. (2020) compared the H-model and P-model for a sugarcane crop in Pernambuco, Brazil, finding the P-model approximately 57 times more conservative than the H-model, though their analysis was deterministic, applied CA to all pesticides regardless of MOA, and adopted a conservative assessment factor of 1000.

With these objectives in mind, the article (1) compares the deterministic P-model (CA for all) with a probabilistic version incorporating parametric uncertainty via Monte Carlo simulation; (2) proposes and implements a legislation-based version of the P-model (CA within HRAC group, IA across groups), also evaluated under Monte Carlo simulation; (3) compares both approaches with the H-model, using a fully traceable, multi-tier-sourced set of $C_{max}$ values and an explicit sensitivity analysis for compounds lacking a compound-specific value; (4) tests the robustness of HRAC group dominance to normalisation by group size; and (5) offers practical recommendations for farmers looking to minimise the grey water footprint.

One more thing is worth saying plainly before the Methods begin. While preparing this version, we audited the entire computational pipeline: every input parameter—applied dose, cultivated area, ecotoxicological endpoints, soil half-life, sorption coefficient, runoff fraction, attenuation factor, maximum acceptable concentration—was retraced to a primary, citable source, and the soil attenuation factor was recomputed from first principles (Eqs. 5–6) rather than assumed. We think this kind of transparency is not optional—it is what makes the comparison worth trusting.

**Table 1:** Complete input dataset for the 17 herbicides evaluated in this study (source: Paraíba et al. (2014), Tables 1 and 2; full citation trail for $C_{max}$ in Supplementary Table S1)

Herbicide	HRAC	$A_D$	$A_C$	EC$_{50}^{min}$	$t_{1/2}$	$K_{oc}$	α	*AF*	$C_{max}$
	group	(kg ha$^{-1}$)	(ha)	(mg L$^{-1}$)	(day)	(L kg$^{-1}$)		(recomputed)	(mg L$^{-1}$)
Ametryn	C1	2.23	$1.88\times 10^{6}$	0.0037	60	300	0.0110	$3.2\times 10^{-18}$	0.0014
Amicarbazone	C1	1.00	$3.36\times 10^{4}$	0.084	54	37	0.0470	$8.7\times 10^{-6}$	7.3
Carfentrazone	E2	0.04	$3.36\times 10^{4}$	0.0127	3	750	0.0001	∼0	0.118
Clomazone	F4	1.00	$1.52\times 10^{6}$	3.5	24	300	0.0070	$1.9\times 10^{-44}$	0.35
Diuron	C1	1.83	$1.00\times 10^{6}$	0.0024	90	480	0.0080	$2.7\times 10^{-18}$	0.0002
Glyphosate	G	1.62	$9.22\times 10^{5}$	1.3	47	24000	0.0001	∼0	0.065
Hexazinone	C1	0.29	$8.51\times 10^{5}$	0.0068	90	54	0.0450	$2.6\times 10^{-4}$	17
Imazapic	B	0.22	$6.69\times 10^{5}$	0.0523	90	1	0.1130	$1.4\times 10^{-2}$	0.0001
Imazapyr	B	0.33	$5.02\times 10^{5}$	12.2	90	100	0.0300	$7.9\times 10^{-6}$	0.036
Isoxaflutole	F1	0.16	$3.03\times 10^{5}$	0.14	100	400	0.0100	$3.6\times 10^{-14}$	0.0001
Metribuzin	C1	1.58	$2.78\times 10^{5}$	0.0081	40	60	0.0310	$3.0\times 10^{-9}$	0.001
Oxyfluorfen	E2	2.00	$1.32\times 10^{4}$	0.0003	35	5000	0.0010	∼0	0.037
Pendimethalin	E	1.38	$2.53\times 10^{5}$	0.0054	90	5000	0.0010	∼0	0.0063
Sulfentrazone	E2	0.70	$7.08\times 10^{4}$	0.031	540	887	0.0060	$7.1\times 10^{-6}$	0.0001
Tebuthiuron	C1	1.00	$6.06\times 10^{4}$	0.05	360	80	0.0440	$7.7\times 10^{-2}$	0.0001
Trifloxysulfuron	B	0.04	$5.31\times 10^{4}$	0.0065	78	1	0.1100	$7.2\times 10^{-3}$	0.0001
Trifluralin	E	0.80	$3.32\times 10^{4}$	0.0007	60	8000	0.0001	∼0	0.0003

## Materials and methods

### Dataset

We used the complete dataset from the original study by Paraíba et al. (2014), comprising 17 herbicides registered for sugarcane in Brazil. The data include recommended dose ($A_D$, kg ha$^{-1}$), application area ($A_C$, ha), EC$_{50}$ for algae, daphnids, and fish (mg L$^{-1}$), soil half-life ($t_{1/2}$, day), the soil organic-carbon sorption coefficient ($K_{oc}$, L kg$^{-1}$), and the runoff fraction (α, estimated by the Soilfug model in the original source). The complete, fully sourced input dataset is reported in Table 1 below, with a tier-by-tier documentation of every $C_{max}$ value given in “Comparison with the H-model and $C_{max}$ sourcing” and reproduced in full, including primary-source citations, in Supplementary Table S1. We retain this 2014 dataset, rather than compiling a new one, because it is the only fully paired source, to our knowledge, that reports application area, dose, and ecotoxicological endpoints for the same 17-herbicide sugarcane programme, allowing the CA and IA comparisons developed here to be attributed to the choice of mixture model rather than to differences in underlying data; the same dataset is also what Barreto et al. (2020) used for the comparison we build on. We flag two respects in which it may no longer fully reflect current practice. First, cultivated area ($A_C$) was compiled from 2012 CONAB figures and herbicide-specific market share at that time; sugarcane area and the relative use of individual herbicides have likely shifted since, so the absolute $V_{GW}$ totals reported here should be read as indicative of relative hazard rather than as a current-year inventory. Second, the regulatory status of some compounds may have changed: to our knowledge, none of the 17 herbicides has since been banned for sugarcane use in Brazil, but registration and use restrictions are periodically revised by national authorities, and we did not re-verify current registration status for each compound as part of this study. Updating $A_C$, market share, and registration status against present-day agricultural and regulatory records is a natural next step we recommend for future applications of this framework and does not affect the validity of the CA-versus-IA comparison itself, which depends on toxicological and fate parameters rather than on cultivated area.

Herbicides were classified by mode of action using the **HRAC** (Herbicide Resistance Action Committee) system. We retain the legacy letter-based group codes (e.g. C1, E2) used in Paraíba et al. (2014); Barreto et al. (2020), rather than the numeric codes HRAC introduced more recently, purely for direct comparability with the dataset’s original source and with the sugarcane-specific literature this study builds on; the two systems are a one-to-one relabelling of the same mechanistic groups, so the choice affects no result. Herbicides in the same HRAC group share the same specific biochemical site of action (e.g. photosynthesis inhibition, amino-acid synthesis inhibition, cell-division disruption) and are, on that basis, treated here as combining additively (Concentration Addition) when applied together, whereas herbicides in different HRAC groups act through unrelated mechanisms and are treated as combining via Independent Action—this distinction is the biological basis for the CA-within-group, IA-across-group framework developed in “Model equations”. We note, however, that sharing a physiological target is not itself a guarantee of strictly additive joint action: additivity also depends on each compound’s precise mechanism upstream and downstream of that target, which is frequently uncharacterised at the level of detail this would require. Concentration addition is nonetheless the standard, conservative default assumption for compounds sharing a target site when the finer mechanistic details are unknown (Backhaus et al. 2003), which is the position we adopt here.

### Model equations

*A nomenclature table listing every symbol used here, with definitions and units, is given in* the Appendix (Table 9).

The total grey water volume of the pesticide mixture under CA is obtained from the condition that the summed hazard quotient of the mixture equals unity,

2 $$\begin{aligned} \sum _{i=1}^{n} \frac{PEC_i}{PNEC_i} = 1 \end{aligned}$$

where $PEC_i$ is the predicted environmental concentration and $PNEC_i$ the predicted no effect concentration of pesticide *i* in water. $PNEC_i$ is derived from the lowest EC$_{50}$ among the three trophic levels tested:

3 $$\begin{aligned} PNEC_i = \frac{EC_{50,i}^{min}/1000}{ASF} \end{aligned}$$

with assessment factor ASF=100 (EEC 2003). The pesticide mass reaching water bodies is

4 $$\begin{aligned} M_i = \alpha _i A_{C,i} A_{D,i} + (1-\alpha _i) A_{C,i} A_{D,i} AF_i \end{aligned}$$

where $AF_i$ is the pesticide attenuation factor from soil surface to groundwater,

5 $$\begin{aligned} AF_i = \exp \left( -\frac{k_i \, z \, R_{F,i} \, \theta _{fc}}{J_w}\right) , \qquad k_i = \frac{\ln 2}{t_{1/2,i}} \end{aligned}$$

and the retardation factor is

6 $$\begin{aligned} R_{F,i} = 1 + \frac{\rho _s \, f_{oc} \, K_{oc,i}}{\theta _{fc}} \end{aligned}$$

Soil parameters follow Paraíba et al. (2014): depth z=2.0 m, bulk density $\rho _s = 1.5$ kg L$^{-1}$, organic-carbon fraction $f_{oc} = 0.003$, field capacity $\theta _{fc} = 0.25$, net recharge $J_w = 9.18\times 10^{-4}$ m day$^{-1}$. $AF_i$ **is recomputed here directly from** Eqs. 5–6**using the verified** $K_{oc}$ **and** $t_{1/2}$ **for each compound** (Supplementary Table S1). This recomputation yields *AF* values spanning many orders of magnitude (from ∼0 for strongly-sorbed, persistent compounds such as glyphosate, to $7.7\times 10^{-2}$ for tebuthiuron, the most leaching-prone compound in the set), consistent with known environmental behaviour of these herbicides.

The grey water volume of the mixture (CA, deterministic) is

7 $$\begin{aligned} V_{GW}^{PM} = \sum _{i=1}^{n} \frac{M_i}{PNEC_i} \end{aligned}$$

where $PNEC_i$ (Eq. 3) is already expressed in kg m$^{-3}$—the factor of $1000^{-1}$ in its definition converts $EC_{50,i}^{min}$ from mg L$^{-1}$ directly to kg m$^{-3}$ (numerically identical to g m$^{-3}$)—so $M_i$ (kg) divided by $PNEC_i$ (kg m$^{-3}$) already yields cubic metres without any further conversion. The individual contribution is $V_{GW,i} = M_i/PNEC_i$, the per-hectare value is $V_{GW,i}^{ha} = V_{GW,i}/A_{C,i}$, and the score is $r_i = \log _{10}(V_{GW,i}^{ha})$.

**Fig. 1:**
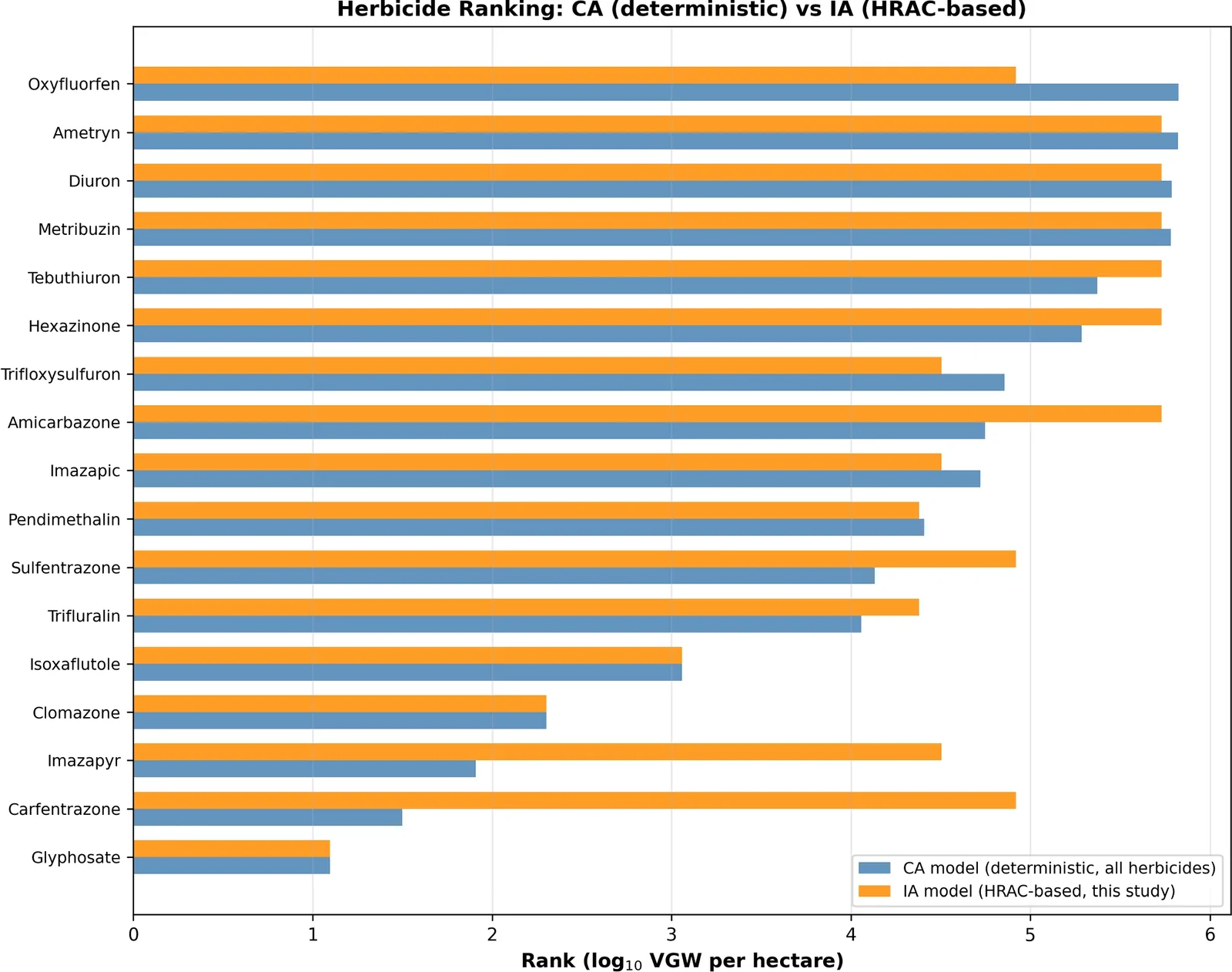
Herbicide score comparison: CA model (deterministic, all herbicides; blue bars) vs. IA model (HRAC-based, this study; orange bars). Lower score values indicate lower grey water footprint per hectare. Generated directly from the verified dataset (Supplementary Table S1); not a placeholder or illustrative figure

#### Score definition under CA versus IA

We deliberately call this metric a *score* rather than a “rank” throughout the manuscript: $\log _{10}(V_{GW}^{ha})$ is a continuous hazard index attached to each herbicide or group, not an ordinal position, and reserving “rank” for the ordinal sense (as in the mean/median group orderings of Table 6) avoids the ambiguity of using the same word for two different things. Two distinct scores are reported for each herbicide throughout this manuscript, and they answer two different questions. The *CA score*, $r_i^{CA} = \log _{10}(V_{GW,i}^{ha})$ as defined above, answers: “if this herbicide were applied alone (or assessed independently of its mixture partners), how large is its own grey water footprint per hectare?” It is computed individually for each of the 17 herbicides from its own $M_i$ and $PNEC_i$, with no reference to HRAC grouping. The *IA score*, by contrast, answers a different question: “given that this herbicide is applied as part of a mixture, and that compounds sharing its mode of action combine additively (CA) while different modes of action combine via Independent Action, how large is the grey water footprint actually attributable to its HRAC group as a whole?” It is defined as $r_g^{IA} = \log _{10}(V_{GW}^{group,g,ha})$ (Eq. 11 below, evaluated per hectare using the group’s combined cultivated area), and *every herbicide in group g is assigned the same IA score,* $r_g^{IA}$ , regardless of its own individual CA score. This is why the two scores differ in Fig. 1 for herbicides belonging to multi-member groups (e.g. carfentrazone, group E2, three members): its CA score (1.50) reflects only its own toxicity and use pattern, while its IA score (4.92) reflects the combined footprint of the entire group it shares a mode of action with—the practically relevant quantity once mixture use is considered, since under CA-within-group the group’s members are assumed toxicologically additive. Herbicides that are the sole member of their HRAC group (e.g. glyphosate, group G) necessarily have identical CA and IA scores, since there is no group to combine with. We deliberately compare an individual herbicide’s own CA score against its group’s IA score, rather than against an IA score computed for that herbicide in isolation, because the practically relevant question for a farmer choosing among herbicides is the footprint actually attributable to the compound once it is used in the multi-mode-of-action programmes that are standard practice in sugarcane (“Introduction”); “Group-size robustness and the role of individual compounds within a group” complements this comparison with a group-size-normalised analysis (Table 6) confirming that group-level scores are not an artefact of how many compounds happen to share a mode of action.

For the HRAC-based IA extension, herbicides within the same HRAC group are assumed to share a common physiological mode of action and therefore combine via concentration addition, giving a group-level hazard quotient at dilution volume *V*:

8 $$\begin{aligned} HQ_g(V) = \sum _{i \in g} \frac{M_i}{V \cdot PNEC_i} \end{aligned}$$

Different HRAC groups act through unrelated physiological mechanisms and are therefore combined across groups using Independent Action (Bliss 1939), under which the combined hazard fraction of the full mixture is

9 $$\begin{aligned} HQ_{mix}(V) = 1 - \prod _{g=1}^{m} \left( 1 - HQ_g(V)\right) \end{aligned}$$

Because $HQ_{mix}(V)=1$ whenever *any* single group’s own hazard quotient reaches 1, the equation is satisfied by up to *m* distinct values of *V* (one for each group); we define the total grey water volume under IA, $V_{GW}^{total}$, as the *greatest* of these—the value below which at least one group’s own dilution requirement is still unmet, and above which every group’s hazard quotient is below 1. Note that each group-level hazard quotient takes the simple form $HQ_g(V) = V_{GW}^{group,g}/V$, where $V_{GW}^{group,g}:= \sum _{i \in g} M_i/PNEC_i$ is exactly the group’s own within-group CA grey water volume (Eq. 7 applied to the subset of compounds in group *g*)—i.e. $V_{GW}^{group,g}$ is, by definition, the dilution volume at which group *g*’s own hazard quotient equals exactly 1.

**Table 2:** Probability density functions used in the Monte Carlo simulation

Parameter	Distribution	Probability density function
EC$_{50}$	Lognormal	$f(x) = \dfrac{1}{x\sigma \sqrt{2\pi }}\exp \left( -\dfrac{(\ln x - \mu _{\ln })^2}{2\sigma ^2}\right)$, CV=30%
α (runoff)	Beta	$f(x) = \dfrac{x^{a-1}(1-x)^{b-1}}{B(a,b)}$, x∈[0,1], CV=40%
*AF* (attenuation)	Trunc. normal	$f(x) = \dfrac{1}{\sigma }\dfrac{\phi \left( \frac{x-\mu }{\sigma }\right) }{\Phi \left( \frac{1-\mu }{\sigma }\right) -\Phi \left( \frac{-\mu }{\sigma }\right) }$, x∈[0,1], CV=35%

This observation yields a short, exact derivation of $V_{GW}^{total}$, rather than a numerically solved approximation. Evaluate $HQ_{mix}(V)$ at $V = \max _g(V_{GW}^{group,g})$, and let $g^*$ denote the group achieving this maximum. By the definition above, $HQ_{g^*}(V) = V_{GW}^{group,g^*}/V = 1$ exactly, so the factor $(1-HQ_{g^*}(V))$ in Eq. 9 is exactly zero—and therefore, the entire product $\prod _{g=1}^m (1-HQ_g(V))$ is exactly zero, *regardless of the hazard quotients of every other group*. Consequently,

10 $$\begin{aligned} HQ_{mix}\left( \max _g V_{GW}^{group,g}\right) = 1 - 0 = 1 \quad \text {identically, for any dataset.} \end{aligned}$$

This proves that

11 $$\begin{aligned} V_{GW}^{total} = \max _g \left( V_{GW}^{group,g} \right) , \qquad V_{GW}^{group,g} = \sum _{i \in g} \frac{M_i}{PNEC_i} \end{aligned}$$

is not an approximation but the *exact* solution to Eq. 9 under this hazard-quotient formulation of Independent Action—specifically, the largest of the (up to *m*) roots at which one group’s hazard quotient crosses 1, which is also the physically relevant root (for *V* infinitesimally above this value, every group’s hazard quotient is below 1 and $HQ_{mix}(V)<1$; for *V* below it, the dominant group’s quotient exceeds 1, and the product formulation is no longer interpretable as a combination of hazard fractions in [0, 1]). We confirmed this identity numerically using the deterministic point estimates ($HQ_{mix}$ evaluated at $V=\max _g V_{GW}^{group,g}$ equals 1 to machine precision), and it holds, by the algebraic argument above, for every Monte Carlo iteration individually—so no approximation error propagates into the probabilistic IA results of “HRAC group contributions under the IA model”.

### Monte Carlo simulation parameters

Parametric uncertainty was incorporated via three probability distributions, summarised in Table 2 below, each parameterised by its empirical mean μ and an assigned coefficient of variation *CV*:

where, for the lognormal distribution, $\sigma ^2 = \ln (1+CV^2)$ and $\mu _{\ln } = \ln (\mu ) - \sigma ^2/2$ so that the distribution’s mean equals the empirical point estimate μ; for the Beta distribution, shape parameters *a* and *b* are obtained from μ and variance $\sigma ^2=(\mu \cdot CV)^2$ via $a = \mu \left( \frac{\mu (1-\mu )}{\sigma ^2}-1\right)$, $b=(1-\mu )\left( \frac{\mu (1-\mu )}{\sigma ^2}-1\right)$; and for the truncated-normal distribution, ϕ and Φ are the standard normal density and cumulative distribution functions, with untruncated standard deviation $\sigma = \mu \cdot CV$ and the distribution truncated to [0, 1]. The distribution family was chosen to match each parameter’s support and typical empirical behaviour: EC$_{50}$ values, like most ecotoxicological endpoints, are strictly positive and characteristically right-skewed across species and compounds, for which the lognormal is the standard choice in probabilistic risk assessment; α and *AF* are both bounded fractions on [0, 1], for which the Beta and truncated-normal families (respectively) provide flexible, appropriately bounded shapes without the lognormal’s unbounded right tail. $C_{max}$ is treated as fixed (deterministic), consistent with Paraíba et al. (2014); Hoekstra et al. (2011). All Monte Carlo sampling and downstream calculations were implemented in Python 3 (NumPy, SciPy, Pandas; “Computational implementation” gives full computational details). N=5000 iterations were used for the main probabilistic analyses (random seed fixed at 42 for reproducibility), and $N=10{,}000$ for the post hoc multimodality analysis (“Multimodality analysis of the IA model under Monte Carlo”; seed 123).

#### Purpose of the probabilistic evaluation

The deterministic point estimates of “Analysis 1: deterministic P-model (CA for all)” give a single value for each herbicide’s grey water footprint, but every input other than $A_D$, $A_C$, and $C_{max}$ is itself an estimate subject to measurement and inter-laboratory variability. The Monte Carlo analysis propagates that input uncertainty through Eqs. 3–7 to quantify how much confidence the resulting $V_{GW}$ and *R* values warrant, and, in “Multimodality analysis of the IA model under Monte Carlo”, whether the qualitative conclusion (which HRAC group dominates) is itself uncertain or robust. We restrict the probabilistic treatment to EC$_{50}$, α, and *AF* because these are the parameters with a documented, quantifiable source of variability at the compound level (inter-study variation in toxicity testing; site-dependent runoff and leaching behaviour), whereas $A_D$ and $A_C$ are registration and land-use figures reported as fixed values by the source (CONAB 2012; Paraíba et al. 2014) rather than as measured quantities with an associated uncertainty distribution; treating them probabilistically would require an assumed variability that we have no empirical basis for, whereas the fate and toxicity parameters do.

#### Independence assumption

In the main probabilistic analyses (“Analysis 1: deterministic P-model (CA for all)”–“Analysis 3: HRAC-based P-model (CA within group, IA across groups)”), all parameters—EC$_{50}$, α, and *AF*—are sampled independently of one another and of the applied dose $A_D$ and cultivated area $A_C$ (which are treated as fixed, since they reflect agronomic registration data rather than environmentally variable quantities). We state this independence assumption explicitly because, in real field systems, α (runoff) and *AF* (leaching/attenuation) plausibly co-vary with local soil and rainfall conditions and could in principle be correlated with each other for a given site (e.g. a wetter, more permeable soil could simultaneously increase both runoff and leaching relative to a drier, less permeable one, or could trade off between them depending on the dominant transport pathway). Toxicity endpoints (EC$_{50}$) are a separate biological axis (laboratory bioassay results on algae/daphnids/fish) with no mechanistic link to a compound’s soil-transport behaviour, so independence between EC$_{50}$ and the fate parameters is a more defensible default. To assess the sensitivity of our results to the independence assumption between α and *AF* specifically—the pair most plausibly correlated through shared site hydrology—“Sensitivity analysis: α–*AF* parameter correlation” introduces an exploratory Gaussian-copula sensitivity analysis at ρ=0.2; “Sensitivity of uncertainty estimates to parameter correlation” reports that this induces a negligible change in the total $V_{GW}^{PM}$ estimate (Table 7).

### Analysis 1: deterministic P-model (CA for all)

This analysis replicates the model of Paraíba et al. (2014) using Eqs. 3–7 without parametric uncertainty, using the verified point estimates from Supplementary Table S1.

### Analysis 2: probabilistic P-model (CA for all)

This analysis applies the distributions of “Monte Carlo simulation parameters” to generate 95% confidence intervals for $V_{GW}^{ha}$, total $V_{GW}^{PM}$, and coefficients of variation.

### Analysis 3: HRAC-based P-model (CA within group, IA across groups)

This analysis implements Eqs. 8–11 under the same Monte Carlo framework, using paired draws from the Analysis 2 samples (i.e., the same iteration’s EC$_{50}$/α/*AF* draws are reused across the group-aggregation step, preserving the natural correlation structure that arises from shared distributional assumptions within an iteration).

### Comparison with the H-model and $C_{max}$ sourcing

For each herbicide, $V_{GW}^{H}$ was calculated from Eq. 1. $C_{max}$ values were compiled from five source tiers, fully documented in Supplementary Table S1: (1) CONAMA Resolution n.^o^ 357/2005 (glyphosate); (2) Barreto et al. (2020) (diuron, imazapyr, metribuzin); (3) US-EPA Aquatic Life Benchmarks, freshwater-vertebrate chronic value (ametryn, amicarbazone, carfentrazone, clomazone); (4) compound-specific US-EPA Endangered Species Act risk-assessment documents, chronic fish NOEC/NOEL (hexazinone, oxyfluorfen, pendimethalin, trifluralin); (5) a generic EU individual-pesticide drinking-water parametric value (Directive (EU) 2020/2184, Annex I, Part B; 0.1 μg L$^{-1}$), applied uniformly to the five remaining compounds (imazapic, isoxaflutole, sulfentrazone, tebuthiuron, trifloxysulfuron) lacking a compound-specific figure. Because the EU maximum residue concentration of 0.1 μg L$^{-1}$ per individual pesticide in drinking water is a conservative regulatory default rather than a toxicological endpoint, and because one such compound (imazapic) was found to dominate the aggregate H-model total disproportionately to its measured toxicity (“Comparison of total grey water footprints”), we report $R = V_{GW}^{P}/V_{GW}^{H}$ both for the full 17-compound set and for a restricted 12-compound subset using only tiers 1–4.

### Robustness analysis: normalisation by HRAC group size

To test whether HRAC group dominance under IA reflects genuinely elevated per-compound hazard or is an artefact of group size (larger groups summing more CA terms), we computed, for each group, both the mean and median per-herbicide $V_{GW}^{ha}$ (deterministic point estimates) and compared the resulting group rankings.

### Sensitivity analysis: α–*AF* parameter correlation

The independence assumption between α and *AF* was tested using a Gaussian copula (exploratory ρ=0.2) reproducing each compound’s marginal Beta(α) and truncated-normal(*AF*) distributions while inducing positive rank correlation, applied to all 17 compounds at the total-mixture level.

### Computational implementation

All calculations were implemented in Python 3 (NumPy, SciPy, Pandas, Matplotlib). Complete source code and the verified input dataset are available as supplementary material.

## Results

### HRAC group contributions under the IA model

The total grey water volume for the complete mixture under IA was $V_{GW}^{PM,IA} = 2.27\times 10^{12}$ m$^3$ (95% CI: 1.23–$4.29\times 10^{12}$)—by the identity established in Eq. 11 (“Model equations”), this equals the largest of the seven within-group CA totals—dominated by group C1 (PSII inhibitors), which contributes $5.37\times 10^{5}$ m$^3$ ha$^{-1}$. Table 3 summarises the HRAC group contributions, including the group-level *R* ratio against the H-model.

**Table 3:** HRAC groups under the IA model: contribution to grey water footprint and group-level $R = V_{GW}^P/V_{GW}^H$ ratio (deterministic)

HRAC	MOA	Chemical family	*n*	$V_{GW}^{group}$ (m$^3$ ha$^{-1}$)	IA score	$R_{group}$
C1	PSII inhib.	Urea/triazine/triazinone	6	$5.37 \times 10^{5}$	5.73	15.1
E2	PPO inhib.	Diphenylether	3	$8.30 \times 10^{4}$	4.92	3.3
B	ALS inhib.	Imidazolinone/sulfonylurea	3	$3.20 \times 10^{4}$	4.50	0.2^*^
E	Microtubule inhib.	Dinitroaniline	2	$2.39 \times 10^{4}$	4.38	106.5
F1	HPPD inhib.	Isoxazole	1	$1.14 \times 10^{3}$	3.06	0.1^*^
F4	Bleaching (unk. target)	Isoxazolidinone	1	$2.00 \times 10^{2}$	2.30	10.0
G	EPSPS inhib.	Glycine	1	$1.25 \times 10^{1}$	1.10	5.0

*Groups B and F1 contain compounds carrying the generic EU $C_{max}$ default (imazapic, trifloxysulfuron in B; isoxaflutole in F1), which inflates $V_{GW}^H$ disproportionately for these groups; $R_{group}<1$ should not be read as “H-model more conservative than P-model” but as an artefact of the conservative default (see “Comparison of total grey water footprints” and “Discussion”)

**Table 4:** Probabilistic P-model results (CA for all) for the lowest- and highest-uncertainty herbicides

Herbicide	$V_{GW}^{ha}$ median	95% CI	CV	Interpretation
	(m$^3$ ha$^{-1}$)	(m$^3$ ha$^{-1}$)	(%)	
Tebuthiuron	$2.42\times 10^{5}$	[1.04, 5.07]$\times 10^{5}$	40.2	Lowest uncertainty
Imazapic	$5.23\times 10^{4}$	[1.92, 12.2]$\times 10^{4}$	47.4	–
Trifluralin	$1.12\times 10^{4}$	[0.38, 2.74]$\times 10^{4}$	49.8	–
Oxyfluorfen	$6.55\times 10^{5}$	[2.16, 16.7]$\times 10^{5}$	52.5	Highest uncertainty

### Probabilistic results (selected herbicides)

Coefficients of variation ranged from 40.2% (tebuthiuron, lowest uncertainty) to 52.5% (oxyfluorfen, highest uncertainty)—a narrow and fairly uniform range, consistent with the fact that the recomputed *AF* values are, for most compounds, negligibly small relative to the runoff term, reducing *AF*’s relative contribution to overall variance. Table 4 reports the extremes.

### Comparison of total grey water footprints

Table 5 compares the total grey water volumes across models. The P-model (CA and IA) produced total estimates of $2.3\times 10^{12}$ m$^3$, essentially independent of whether CA or IA is applied at the aggregate level (because, under IA, the total is set entirely by the dominant group, and that group’s CA-summed value is close to the full-mixture CA sum). The H-model total, $3.23\times 10^{11}$ m$^3$, yields R=7.0 (deterministic, full 17-compound set) to R=18.4 (deterministic, 12-compound subset with fully compound-specific $C_{max}$); under the probabilistic restricted-subset analysis, R=19.6 (95% CI, 12.4–33.5). We report both the full-set and restricted-subset *R* throughout, since the full-set value is materially affected by a single compound (imazapic, see below).

**Table 5:** Comparison of total grey water footprints across models

Model	$V_{GW}^{total}$ (m$^3$)	*R*	*R*
	[95% CI]	(full 17)	(restricted 12)
H-model (Hoekstra)	$3.23\times 10^{11}$	–	–
P-model CA (det.)	$2.26\times 10^{12}$	7.0	18.4
P-model CA (prob., median)	$2.33\times 10^{12}$ [1.29, 4.36]$\times 10^{12}$	7.4 [3.9, 14.3]	19.6 [12.4, 33.5]
P-model IA (HRAC, median)	$2.27\times 10^{12}$ [1.23, 4.29]$\times 10^{12}$	7.3 [3.7, 14.0]	–

Twelve of 17 herbicides (CA, deterministic) showed P-model > H-model. We note that the deterministic CA total ($2.26\times 10^{12}$ m$^3$) is close to the total originally reported by Paraíba et al. (2014) themselves ($2.36\times 10^{12}$ m$^3$), confirming that the present recalculation faithfully reproduces the foundational model when restricted to its original (CA-for-all) formulation.

#### On the imazapic distortion

A single compound, imazapic, contributes 51.5% of the total deterministic $V_{GW}^H$ across all 17 herbicides—not because it is unusually toxic, but because it carries the generic EU $C_{max}$ default (0.0001 mg L$^{-1}$, the most conservative tier in our sourcing hierarchy) combined with a comparatively large cultivated area and the highest α in the dataset. The five EU-generic-default compounds together account for 62.9% of total $V_{GW}^H$. This is the principal reason the restricted-subset *R* (18.4–19.6) differs so markedly from the full-set *R* (7.0–7.4); we present both, with the restricted subset as our preferred estimate of the true *P*/*H* ratio, while retaining the full-set value as a conservative (precautionary) bound that follows directly from treating an unknown $C_{max}$ as protectively as EU drinking-water policy does.

**Fig. 2:**
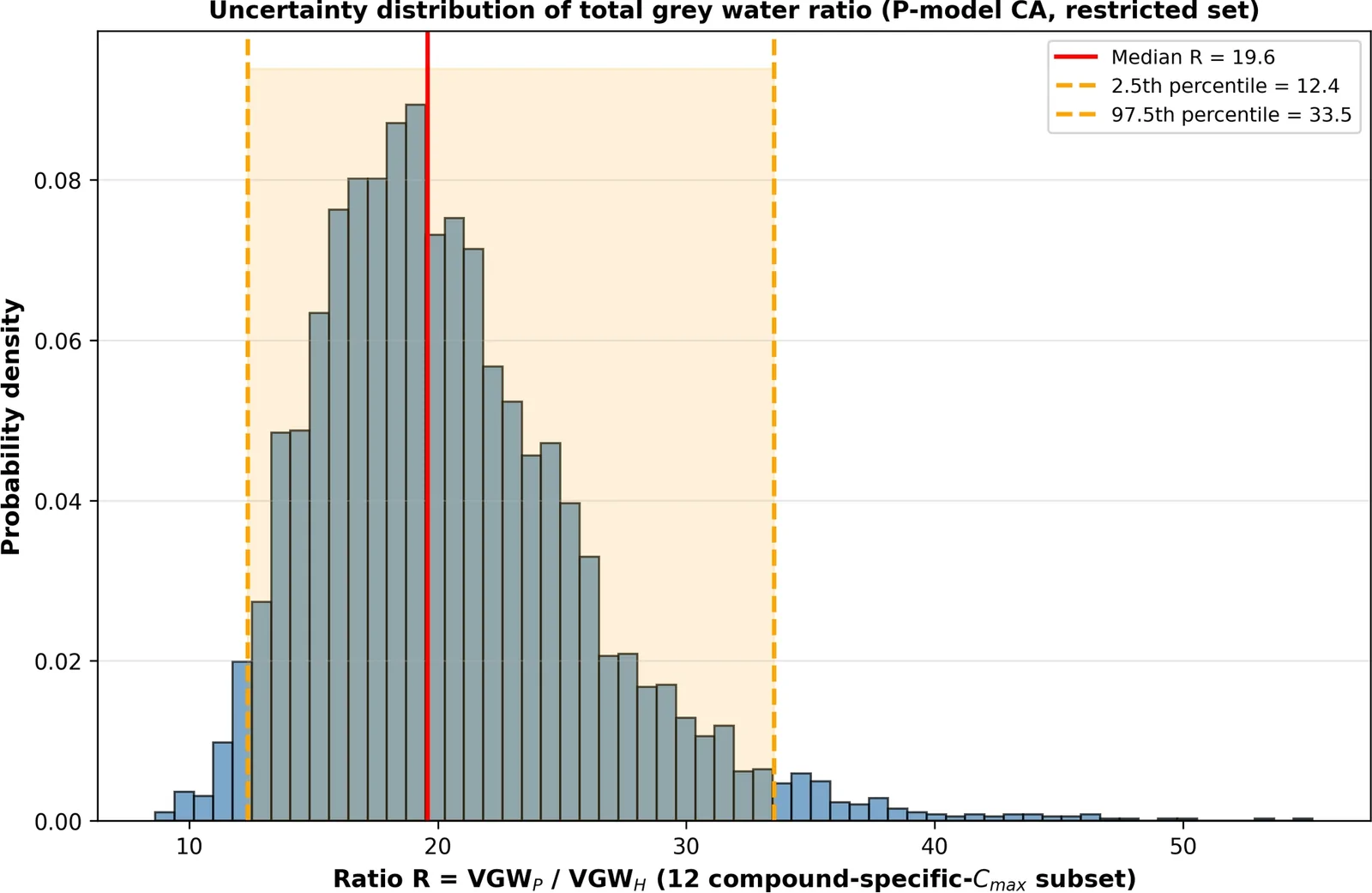
Uncertainty distribution of the total grey water ratio $R = V_{GW}^{P}/V_{GW}^{H}$ (P-model CA vs. H-model, restricted 12-compound subset). Generated directly from the Monte Carlo output of this analysis (*N*=5000); not a synthetic approximation

### Ranking comparison: CA versus IA

Figure 1 presents the herbicide scores under both models (both computed from the deterministic point estimates of Supplementary Table S1, not Monte Carlo medians). The most dramatic changes occurred for carfentrazone (group E2), whose score rose from 1.50 (CA) to 4.92 (IA) because it inherits its group’s elevated score, and imazapyr (group B), rising from 1.91 to 4.50. Conversely, glyphosate (group G, single herbicide) maintained its low score (1.10) under both models, since a single-member group is unaffected by the CA-within-group step. For a few herbicides (e.g. trifloxysulfuron, group B), the CA bar in Fig. 1 exceeds the IA bar for their own group: this is not an inconsistency but a consequence of the per-hectare normalisation—the individual CA score divides the herbicide’s own footprint by its own cultivated area, whereas the group IA score divides the group’s combined footprint by the group’s combined cultivated area, which can be substantially larger; a herbicide applied over a comparatively small area within a group dominated by larger-area members can therefore show a higher individual CA score than the group’s own IA score.

**Fig. 3:**
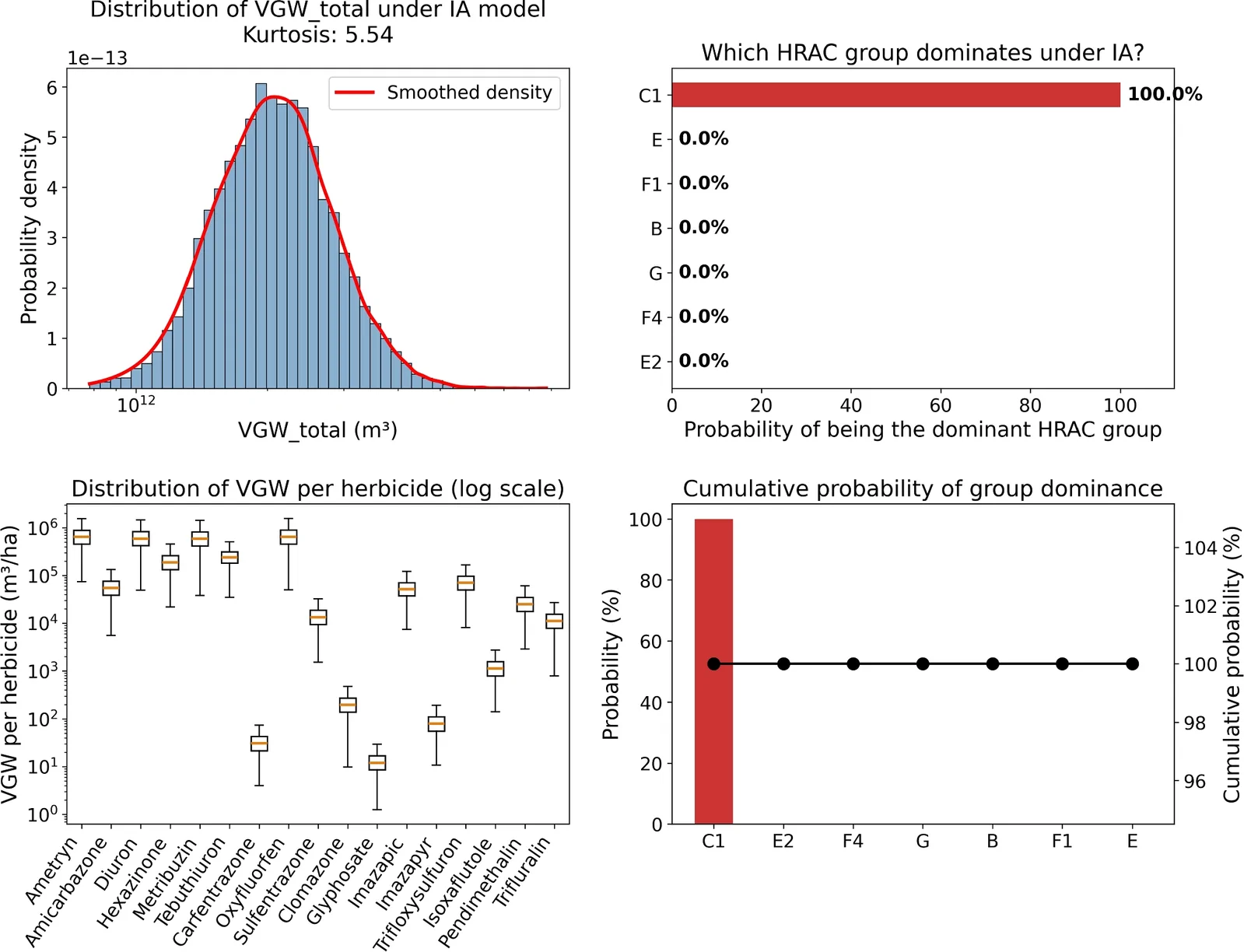
Multimodality analysis of the total grey water footprint ($V_{GW}^{total}$) distribution under the IA model ($N=10{,}000$). Top left: histogram with smoothed density (unimodal). Top right: probability of each HRAC group being the dominant contributor (C1, 100%; all others, 0%). Bottom left: per-herbicide $V_{GW}^{ha}$ distribution (log scale), grouped by HRAC. Bottom right: cumulative probability of group dominance

### Uncertainty distribution of the P/H ratio

Figure 2 shows the probability distribution of $R = V_{GW}^P/V_{GW}^H$ under the probabilistic CA model, restricted-subset analysis (12 compounds with fully compound-specific $C_{max}$). The distribution is right-skewed with median 19.6 and 95% CI [12.4, 33.5].

### Multimodality analysis of the IA model under Monte Carlo

The post-hoc multimodality analysis ($N=10{,}000$; Fig. 3) shows that HRAC group C1 dominates the grey water footprint in **100%** of iterations. Kurtosis of the $V_{GW}^{total}$ distribution is 5.54, indicating a leptokurtic (sharply peaked, long-tailed) distribution.

#### Interpretation of the bimodality coefficient

To formally test for bimodality (as opposed to relying on visual inspection of Fig. 3a alone), we computed Sarle’s bimodality coefficient, $BC = (\gamma _1^2+1)/\gamma _2$, where $\gamma _1$ is the sample skewness and $\gamma _2$ the sample kurtosis (non-excess/Pearson convention) of the $V_{GW}^{total}$ distribution. For the present distribution ($\gamma _1=1.12$, $\gamma _2=5.54$), BC=0.405. The conventional reference value is 5/9≈0.555, the bimodality coefficient of a uniform distribution: values of *BC* *above* this threshold are taken to suggest bimodality (or, more generally, a distribution flatter/more multi-peaked than uniform), while values *below* it indicate a distribution more sharply single-peaked than uniform, consistent with unimodality. Because BC=0.405<0.555, this is a quantitative confirmation—not merely a visual impression—that the $V_{GW}^{total}$ distribution is unimodal, consistent with the high kurtosis (a single sharp peak, not two or more separated modes) and with the clean 100% dominance of group C1 reported above: if a substantial fraction of iterations had instead been dominated by a second group with a markedly different $V_{GW}^{total}$ scale, the distribution would show two separated peaks, elevated *BC*, and a less-than-100% dominance split, none of which is observed here.

**Table 6:** Normalisation of HRAC group contributions by group size: mean and median per-herbicide $V_{GW}^{ha}$ (deterministic)

HRAC	*n*	Mean $V_{GW}^{ha}$	Median $V_{GW}^{ha}$	Rank	Rank
		(m$^3$ ha$^{-1}$)	(m$^3$ ha$^{-1}$)	(mean)	(median)
C1	6	$3.94\times 10^{5}$	$4.20\times 10^{5}$	1	1
E2	3	$2.27\times 10^{5}$	$1.36\times 10^{4}$	2	4
B	3	$4.15\times 10^{4}$	$5.27\times 10^{4}$	3	2
E	2	$1.85\times 10^{4}$	$1.85\times 10^{4}$	4	3
F1	1	$1.14\times 10^{3}$	$1.14\times 10^{3}$	5	5
F4	1	$2.00\times 10^{2}$	$2.00\times 10^{2}$	6	6
G	1	$1.25\times 10^{1}$	$1.25\times 10^{1}$	7	7

**Table 7:** Sensitivity of total $V_{GW}$ uncertainty to a fixed, exploratory α–*AF* correlation (ρ=0.2), compared to the main independent-parameter simulation

Metric	Independent (ρ=0)	Correlated (ρ=0.2)
Median total $V_{GW}$ (m$^3$)	$2.33\times 10^{12}$	$2.35\times 10^{12}$
95% CI width (m$^3$)	$3.07\times 10^{12}$	$3.05\times 10^{12}$
CI width ratio (correlated/independent)	—	0.994

### Robustness of group dominance to normalisation by group size

Table 6 reports per-herbicide mean and median $V_{GW}^{ha}$ within each HRAC group. Group C1 is the top-ranked group under both normalisations (rank 1 by mean and by median), confirming that its dominance reflects genuinely elevated per-compound hazard rather than the arithmetic effect of summing six CA terms. By contrast, group E2 ranks 2nd by mean but only 4th by median—a large divergence explained by a single high-impact compound, oxyfluorfen ($V_{GW}^{ha}=6.67\times 10^{5}$), against much lower values for carfentrazone (31.5) and sulfentrazone ($1.36\times 10^{4}$).

### Sensitivity of uncertainty estimates to parameter correlation

Table 7 compares the independent-parameter and ρ=0.2 correlated (α–*AF*, Gaussian copula) Monte Carlo scenarios. The median total $V_{GW}^{PM}$ changed by 0.66%, and the 95% CI width *narrowed* slightly (ratio 0.994) under correlation, attributable to the recomputed *AF* values being, for most compounds, vanishingly small and therefore contributing little incremental variance regardless of their correlation with α.

**Table 8:** Farmer decision table: how to minimise the grey water footprint, based on the fully re-verified dataset

Recomm	HRAC	Herbicide(s)	$V_{GW}^{ha}$ (m$^3$ ha$^{-1}$)	Note
Prefer	G	Glyphosate	$1.25\times 10^{1}$	Lowest footprint in the dataset
Prefer	F4	Clomazone	$2.00\times 10^{2}$	—
Prefer	F1	Isoxaflutole	$1.14\times 10^{3}$	$C_{max}$ is the generic EU default; true value may differ
Caution	B	Imazapyr	$8.12\times 10^{1}$	Low individually; group score driven by other members
Caution	B	Imazapic, Trifloxysulfuron	5.3–$7.2\times 10^{4}$	$C_{max}$ is the EU default for imazapic; group H-model total disproportionately affected (Implications of the generic EU $C_{max}$ default and the imazapic finding)
Caution (low ind. risk)	E2	Carfentrazone, Sulfentrazone	31.5–$1.4\times 10^{4}$	Group score driven primarily by oxyfluorfen, not these compounds
Avoid individually	E2	Oxyfluorfen	$6.67\times 10^{5}$	Highest individual value in dataset; principal driver of E2’s footprint
Caution	E	Pendimethalin, Trifluralin	1.1–$2.6\times 10^{4}$	—
Avoid	C1	Ametryn, diuron, metribuzin, hexazinone, tebuthiuron, amicarbazone	$5.6\times 10^{4}$–$6.6\times 10^{5}$	All six members elevated; dominance confirmed by Table 6 and by 100% MC dominance (“Multimodality analysis of the IA model under Monte Carlo”)

## Discussion

### Why mixture toxicology models should inform footprint accounting

At first glance, grey water footprint accounting and mixture ecotoxicology answer different questions. One is a pollution-dilution indicator: how much clean water is needed to bring a pollutant load down to an acceptable concentration. The other describes how multiple stressors combine biologically to produce a joint effect. What ties them together is the *acceptable concentration* itself. The H-model takes that from a regulatory threshold, $C_{max}$; the P-model derives it instead directly from organism-level toxicity data, as *PNEC* (Eq. 3). We do not mean to suggest a strict dichotomy between the two: many regulatory $C_{max}$ values are themselves derived, upstream, from ecotoxicological endpoints, sometimes via the same kind of assessment-factor extrapolation used here for *PNEC*. The practical distinction that matters for this study is narrower: $C_{max}$, once set, is a single per-compound number that carries no explicit rule for how several compounds’ concentrations should be combined in a mixture, whereas building *PNEC* directly from EC$_{50}$ data keeps the mixture-combination question open and lets us apply CA or IA explicitly. A mixture’s footprint is not well-defined under either approach unless one also states how the individual hazard quotients combine—a single pesticide’s dilution volume tells you nothing about what happens when several compounds are present at once, sharing a mechanism of toxic action or not. CA and IA are ecotoxicology’s two limiting-case answers to exactly that question (Loewe 1953; Bliss 1939). Any GWF model applied to a multi-compound exposure is already making a mixture-combination assumption, whether it says so or not—CA-for-all, the original P-model’s choice, is itself one such assumption. What we do here is simply make that assumption conditional on shared mode of action, as standard mixture-toxicology practice recommends, rather than apply it across the board.

To our knowledge, this is the first probabilistic, fully numerically audited comparison of the H-model and the HRAC-based P-model for sugarcane herbicides—though we want to be careful with that claim, because none of the individual pieces are new. The H-model comes from Hoekstra et al. (2011); the CA-based P-model is Paraíba et al.’s (2014) own earlier work, which we extend rather than replace; the CA-within-group/IA-across-group combination rule is standard ecotoxicological practice recommended by European guidance (ECHA 2014; EFSA 2013); and Monte Carlo uncertainty propagation is routine in probabilistic risk assessment. What is new, specifically, is putting these pieces together into one reproducible pipeline, adding a group-size-normalisation robustness check (“Group-size robustness and the role of individual compounds within a group”), and running an explicit sensitivity analysis that isolates the effect of an unverifiable or generic input ($C_{max}$, “Implications of the generic EU $C_{max}$ default and the imazapic finding”). That audit-style transparency, as far as we know, has not accompanied a grey water footprint mixture assessment before—we offer it as an incremental contribution, not a conceptual leap. Barreto et al.’s (2020) central finding, that the P-model is substantially more conservative than the H-model, holds up here too, though at a smaller magnitude: R≈7–20, against Barreto et al.’s own deterministic R≈57. We attribute the difference chiefly to Barreto et al.’s (2020) more conservative assessment factor (1000, versus our ASF=100) and their application of CA across all 17 herbicides regardless of MOA, both of which mechanically inflate their *P*/*H* ratio relative to the HRAC-based framework adopted here.

### The role of mode of action in herbicide scoring

Our results confirm that the choice between CA and IA is not a technical footnote but materially changes herbicide scores. Carfentrazone (group E2, three herbicides) and glyphosate (group G, one herbicide) have similar CA scores (1.50 and 1.10, respectively)—both would appear, under the original Paraíba et al. (2014) CA-for-all formulation, as comparably low-impact choices. Under the IA model, however, carfentrazone “inherits” its group’s elevated score (4.92), becoming a markedly worse choice than glyphosate once it is used alongside other group E2 compounds. This phenomenon follows directly from Eqs. 8–11: when multiple herbicides from the same HRAC group are applied, their CA-summed contribution raises the entire group’s score, and the IA total is set by the single most hazardous group.

### Group-size robustness and the role of individual compounds within a group

The robustness analysis (Table 6) shows that C1’s dominance is not an artefact of containing six members: it is top-ranked whether groups are compared by their summed contribution, their mean per-compound contribution, or their median per-compound contribution. Group E2 tells a different story: its mean-based rank (2nd) is driven almost entirely by oxyfluorfen ($6.67\times 10^{5}$ m$^3$ ha$^{-1}$, itself the single highest individual value in the entire 17-compound dataset), while its other two members, carfentrazone (31.5) and sulfentrazone ($1.36\times 10^4$), are far lower—pulling its median-based rank down to 4th. The practical implication is that group E2 should not be treated as uniformly hazardous: the risk is concentrated almost entirely in oxyfluorfen, and farmer guidance (“Farmer decision table”, Table 8) should reflect that distinction rather than flagging the whole group.

### Implications of the generic EU $C_{max}$ default and the imazapic finding

Five of the 17 herbicides in this study (imazapic, isoxaflutole, sulfentrazone, tebuthiuron, trifloxysulfuron) lack a published, compound-specific maximum acceptable concentration in any of the four primary-source tiers we searched (CONAMA, Barreto et al. 2020, US-EPA Aquatic Life Benchmarks, US-EPA Endangered Species Act risk assessments). Per standard practice when a regulatory $C_{max}$ is genuinely unknown, we applied the EU’s generic individual-pesticide drinking-water parametric value (0.1 μg L$^{-1}$) to these five compounds—the same convention, we note, implicitly underlying the otherwise-uncited “placeholder” value reported by Barreto et al. (2020) for two of these same compounds (“Comparison with the H-model and $C_{max}$ sourcing”).

This convention is conservative by design (it is a human-health drinking-water standard, generally stricter than an aquatic-life-only threshold), and the consequence is that one compound, imazapic, ends up contributing 51.5% of the entire 17-compound $V_{GW}^H$ total—not because it is unusually toxic to aquatic organisms (its EC$_{50}$ values are unremarkable relative to the dataset), but because the conservative default, combined with imazapic’s comparatively large cultivated area and the highest α (runoff fraction) of any compound studied, mechanically inflates its H-model denominator-side mass term. We regard the restricted 12-compound *R* (≈20) as the more defensible estimate of the true *P*/*H* ratio for this reason, while reporting the full-set value (≈7) as an explicitly conservative, precautionary bound. We recommend that future revisions of this analysis seek compound-specific $C_{max}$ values for these five herbicides from primary national or EU pesticide risk-assessment dossiers, which were not accessible within the scope of the present search.

### Farmer decision table

Table 8 translates the per-compound and per-group findings into practical guidance, distinguishing—per “Group-size robustness and the role of individual compounds within a group” above—between group-level avoidance (C1) and compound-specific avoidance within an otherwise moderate group (oxyfluorfen within E2). We present these as a transparent, model-based *screening prioritisation* rather than a definitive prescription: as discussed in “Limitations”, the underlying rankings have not been field-validated, and growers and advisors should treat Table 8 as one input among several (agronomic efficacy, resistance management, cost, and local field conditions) rather than a stand-alone decision rule.

#### Definition of “minimisation” and derivation of the recommendation cut-points

“Minimising the grey water footprint” means selecting, among the herbicides available for a given weed-control purpose, those whose IA score is lowest, since lower $V_{GW,i}^{ha}$ directly reduces the dilution volume required and, when herbicides from different HRAC groups are combined, the IA total is set by the single highest-scoring group present in the mixture (Eq. 11)—so avoiding high-IA-score groups altogether is more effective than any within-group substitution. The three-tier recommendation (prefer/use with caution/avoid) is not based on an arbitrary numerical cutoff: it is derived directly from the two largest discontinuities in the empirical distribution of IA scores across the seven HRAC groups present in this dataset (C1, 5.73; E2, 4.92; B, 4.50; E, 4.38; F1, 3.06; F4, 2.30; G, 1.10). Ordering these seven values, the gaps between consecutive scores are as follows: C1–E2 =0.81, E2–B =0.42, B–E =0.12, E–F1 =1.32 (the largest gap in the dataset), F1–F4 =0.76, F4–G =1.20 (the second-largest gap). These two largest gaps partition the seven groups into three natural clusters: $\{C1, E2, B, E\}$ (IA score ≥4.0, recommended *Avoid* or *Use with caution* depending on within-group compound-level heterogeneity; see “Group-size robustness and the role of individual compounds within a group”), $\{F1, F4\}$ (2.0≤ IA score <4.0, *Use with caution*), and $\{G\}$ (IA score <2.0, *Prefer*). This is why C1 (IA score 5.73) is recommended *Avoid* while E2 (IA score 4.92) is recommended *Use with caution* rather than *Avoid* outright: the two scores are numerically close, and “Group-size robustness and the role of individual compounds within a group” shows that E2’s elevated score is driven almost entirely by a single compound (oxyfluorfen) rather than uniformly across its three members, whereas C1’s elevated score is confirmed, by the group-size-normalisation check in Table 6, to reflect genuinely elevated hazard across all six members. The recommendation therefore combines the gap-based group-level tier with the compound-level robustness check, rather than relying on the group score alone.

### Future perspectives: regulatory support and international relevance

The framework developed here has practical value beyond ranking herbicides within a single Brazilian crop. Many countries, including Brazil, have incomplete freshwater quality standards for pesticides: regulatory maximum acceptable concentrations exist for only a subset of registered active ingredients (in the present dataset, a compound-specific $C_{max}$ could be located for 12 of 17 herbicides; “Analysis 3: HRAC-based P-model (CA within group, IA across groups)”), and almost no jurisdiction sets standards for pesticide *mixtures* rather than single active ingredients. For compounds or mixtures without an established regulatory limit, the P-model framework used here—combining ecotoxicological endpoints, environmental fate parameters, and application rates, with no dependence on $C_{max}$—can support preliminary screening: environmental agencies could use HRAC-based IA rankings of the kind reported in Table 8 to prioritise which pesticides or HRAC groups warrant urgent attention in monitoring programmes, tiered risk assessment, or new standard-setting, particularly for groups (such as C1 in this study) whose footprint is shown to be robust to group-size artefacts rather than an analytical coincidence (“Group-size robustness and the role of individual compounds within a group”).

Nothing in the CA-within-group/IA-across-group framework is specific to herbicides or to sugarcane. The same logic applies directly to fungicides and insecticides, substituting HRAC’s mode-of-action grouping for the analogous classification schemes maintained by the Fungicide Resistance Action Committee (FRAC) and the Insecticide Resistance Action Committee (IRAC), and to any other crop system with a comparable multi-mode-of-action spray programme; the main practical requirement is a dataset pairing application rate, area, and trophic-level EC$_{50}$ values comparable in completeness to Paraíba et al.’s (2014) sugarcane compilation, which is not yet available for most crop-pesticide combinations in Brazil. A recent global assessment reinforces the case for this kind of extension, reporting that pesticide-driven grey water footprints for major crops in China remain substantial and understudied relative to nutrient-driven grey water (Yi et al. 2024), which suggests the gap this framework addresses is not specific to Brazilian sugarcane.

This relevance extends beyond local agricultural management. Pesticide contamination of freshwater is not confined to the field where a compound is applied: rivers, floodplains, wetlands, and groundwater connections can transport pesticides and their residues across long distances, including across national borders in shared river basins. Brazil shares major freshwater systems—including the Amazon basin and several smaller transboundary basins along its borders—with neighbouring countries, and pesticide regulation in one jurisdiction can directly affect water quality and aquatic ecosystems in another. Persistent gaps and inconsistencies in pesticide water-quality regulation across countries have been documented elsewhere: a recent global mapping study found that most countries lack comprehensive pesticide water-quality standards and that regulatory stringency is measurably associated with lower freshwater contamination (Huang and Li 2025), and a companion study proposed a harmonised, health-based regulatory chain framework integrating soil and drinking-water standards across jurisdictions specifically to address this fragmentation (Li and Fantke 2022). A transparent, fully reproducible, mixture-aware screening method of the kind presented here—in which every input is traceable to a cited source (Supplementary Table S1) and the computational pipeline is openly available—could serve as a common technical basis for joint basin management, cross-border monitoring coordination, and harmonised pesticide risk assessment between countries that share freshwater resources, reducing the friction that arises when neighbouring jurisdictions use incompatible or opaque risk-ranking methodologies.

### Limitations

Limitations include the following: (i) five $C_{max}$ values rest on a generic regulatory default rather than a compound-specific toxicological endpoint, with a quantified, reported effect on the aggregate *R* ratio (“Implications of the generic EU $C_{max}$ default and the imazapic finding”); (ii) the IA model assumes complete independence between modes of action; (iii) extrapolation from acute to chronic toxicity using ASF=100 is conservative but standard; (iv) the recomputed attenuation factor, while now traceable to first-principles equations and verified soil parameters, was not independently field-validated for this specific sugarcane production system; (v) **this study presents no ecological field validation of any kind.** All results are model-derived comparisons between two accounting frameworks (H-model and P-model) and have not been checked against measured pesticide concentrations in sugarcane-region surface water or groundwater, nor against observed biological effects in exposed aquatic communities. Consequently, a larger computed grey water footprint value should be read strictly as indicating a higher *modelled* dilution requirement under the stated toxicological and fate assumptions, not as direct evidence of greater real-world ecological harm or of superior predictive accuracy relative to the H-model; the two models could in principle be equally poor (or equally good) predictors of actual field outcomes, a question this study does not and cannot answer. We regard field or monitoring-based validation, ideally using the herbicide concentration and biological-effect data already collected in sugarcane-growing regions of São Paulo state, as the logical and necessary next step before any of the rankings or recommendations reported here (Table 8) are treated as more than a transparent, internally consistent screening prioritisation.

## Conclusion

This study presented a fully numerically audited probabilistic comparison between Hoekstra’s H-model and Paraíba’s P-model, in both its original CA-for-all formulation and an HRAC-based IA formulation. Using verified data for 17 herbicides applied to Brazilian sugarcane, we found that the P-model is more conservative than the H-model, with *R* ranging from 7.0 (full 17-compound set, including a conservative generic default for 5 compounds) to approximately 20 (12-compound subset with fully compound-specific $C_{max}$, our preferred estimate). HRAC group C1 dominates the grey water footprint under IA in 100% of Monte Carlo iterations, and this dominance is robust to normalisation by group size. Herbicide scores depend critically on CA versus IA, with carfentrazone’s score rising from 1.5 to 4.9 once group membership is accounted for. Within group E2, the apparent group-level hazard is concentrated almost entirely in a single compound, oxyfluorfen, illustrating that group-level recommendations should be checked against compound-level data before being applied uncritically. We recommend that certification bodies, regulatory agencies, and farmers consider the P-model with HRAC-based IA as a transparent, traceable screening tool for grey water footprint assessment of pesticides in sugarcane, pending the field or monitoring-based validation discussed in “Limitations”, while continuing to seek compound-specific regulatory thresholds for the small number of herbicides for which none currently exists in the literature we were able to access.

## Supplementary Information

Below is the link to the electronic supplementary material.

Supplementary file 1 (zip 235 KB)

## Data Availability

The complete, fully sourced input dataset (Supplementary Table S1) and the Python source code for all deterministic and probabilistic Monte Carlo calculations are provided as Supplementary Information.

## Appendix. Nomenclature

Table 9 lists every symbol used in Eqs. 1–11, in order of first appearance, with definition and unit.

**Table 9:** Nomenclature: symbols, definitions, and units

Symbol	Definition	Unit
$V_{GW}^{H}$	Grey water volume, H-model (Hoekstra et al. 2011)	m$^3$
α	Runoff fraction: fraction of applied pesticide reaching surface water by runoff	Dimensionless
$A_C$	Cultivated area	ha
$A_D$	Applied (recommended) dose	kg ha$^{-1}$
$C_{max}$	Maximum acceptable pesticide concentration in water (regulatory or screening)	mg L$^{-1}$
$PEC_i$	Predicted environmental concentration of pesticide *i* in water	kg m$^{-3}$
$PNEC_i$	Predicted no effect concentration of pesticide *i* in water	kg m$^{-3}$
$EC_{50,i}^{min}$	Lowest median effective concentration among algae, daphnids, and fish for pesticide *i*	mg L$^{-1}$
*ASF*	Assessment (safety) factor applied to $EC_{50}^{min}$ in deriving *PNEC*	Dimensionless (=100)
$M_i$	Pesticide mass reaching water bodies (runoff + leaching)	kg
$AF_i$	Soil attenuation factor (surface to groundwater) for pesticide *i*	Dimensionless
$k_i$	Soil first-order degradation rate constant, $k_i=\ln 2/t_{1/2,i}$	day$^{-1}$
$t_{1/2,i}$	Soil half-life of pesticide *i*	day
*z*	Soil profile depth (assumed homogeneous)	m
$R_{F,i}$	Retardation factor of pesticide *i* (leaching delay due to sorption)	Dimensionless
$\theta _{fc}$	Soil volumetric water content at field capacity	Dimensionless
$J_w$	Net water recharge rate of the soil	m day$^{-1}$
$\rho _s$	Total soil (bulk) density	kg L$^{-1}$
$f_{oc}$	Soil organic-carbon volumetric fraction	Dimensionless
$K_{oc,i}$	Soil organic-carbon/water partition (sorption) coefficient of pesticide *i*	L kg$^{-1}$
$V_{GW}^{PM}$	Total grey water volume of the mixture, P-model, Concentration Addition	m$^3$
$V_{GW,i}$	Grey water volume attributable to pesticide *i* individually	m$^3$
$V_{GW,i}^{ha}$	Per-hectare grey water volume of pesticide *i*, $=V_{GW,i}/A_{C,i}$	m$^3$ ha$^{-1}$
$r_i$ (or $r_i^{CA}$)	Individual CA score of pesticide *i*, $=\log _{10}(V_{GW,i}^{ha})$	Dimensionless (log scale)
$HQ_g(V)$	Group-level hazard quotient of HRAC group *g* at dilution volume *V*	Dimensionless
$HQ_{mix}(V)$	Combined hazard fraction of the full mixture (independent action) at *V*	Dimensionless
$V_{GW}^{group,g}$	Within-group (CA) grey water volume of HRAC group *g*	m$^3$
$V_{GW}^{total}$	Total grey water volume of the mixture under the HRAC-based IA model	m$^3$
$r_g^{IA}$	IA score of HRAC group *g* (shared by all its member herbicides), $=\log _{10}(V_{GW}^{group,g,ha})$	Dimensionless (log scale)
$V_{GW}^{H}$ (per compound)	H-model grey water volume of an individual pesticide, $=1000\,\alpha A_C A_D/C_{max}$	m$^3$
*R*	Ratio of P-model to H-model total grey water volume, $V_{GW}^{P}/V_{GW}^{H}$	Dimensionless
*CV*	Coefficient of variation assigned to a Monte Carlo input distribution	Dimensionless
*N*	Number of Monte Carlo iterations	Dimensionless
ρ (copula)	Gaussian-copula rank correlation parameter, α–*AF* sensitivity analysis	Dimensionless
*BC*	Sarle’s bimodality coefficient, $=(\gamma _1^2+1)/\gamma _2$	Dimensionless
$\gamma _1$, $\gamma _2$	Sample skewness and (Pearson, non-excess) kurtosis	Dimensionless
