# Supplementary material for: Probabilistic comparisons of grey water footprint models for herbicide mixtures in Brazilian sugarcane
Source: Environ Sci Pollut Res Int. 2026 Aug 12;33(26):13415–31. doi: 10.1007/s11356-026-38125-9 (PMC13503512; doi:10.1007/s11356-026-38125-9)
Supplement: Supplementary file 1 [file 11356_2026_38125_MOESM1_ESM.zip › Table_S1_supplementary.pdf]

Supplementary Table S1

Complete input dataset for the 17 herbicides used in the deterministic and probabilistic grey water footprint (GWF) calculations

This table reports the complete herbicide-level input dataset underlying all deterministic and probabilistic results presented in the main text. Applied dose (A_D), cultivated area (A_C), EC_{50} for algae, daphnids and fish, soil half-life ($t_{1/2}$), and the soil organic-carbon sorption coefficient (K_{oc}) are reproduced from Paraíba et al. (2014), Table 1 (data originally compiled from the US-EPA Pesticide Ecotoxicity Database and from Hornsby et al., 1996). The runoff fraction (α, estimated by the Soilfug model) is reproduced from Paraíba et al. (2014), Table 2. The attenuation factor (AF) is recomputed from first principles (main text, Eqs. 5–6) rather than taken from the original source. HRAC mode-of-action group assignment follows the classification used throughout the present study (main text, Table 1).

Note on C_{max}. All 17 herbicides are populated using five source tiers, documented in full in the main text (Section 2.7):

1. Glyphosate (0.065 mg L^{-1}) – CONAMA Resolution n.° 357/2005, Art. 14–15 (freshwater Classes 1–2 standard), independently cross-checked against Barreto et al. (2020), Table 1, which reports the identical value.

2. Ametryn (0.0014 mg L^{-1}) – groundwater acceptable-concentration limit from the US-EPA Reregistration Eligibility Decision (RED) for ametryn, as cited directly in Paraíba et al. (2014) within their own H-model worked example comparing the P-model and H-model for this compound; preferred here over the US-EPA Aquatic Life Benchmark value (freshwater-vertebrate chronic, 0.7 mg L^{-1}) because it is the value the foundational paper itself verified and used.

3. Diuron (0.0002), Imazapyr (0.036), Metribuzin (0.001) – Barreto et al. (2020), Table 1 (joint CONAMA/US-EPA citation, not attributable per compound).

4. Amicarbazone (7.3), Carfentrazone (0.118), Clomazone (0.35) – US-EPA Office of Pesticide Programs, Aquatic Life Benchmarks table, freshwater-vertebrate chronic value.

5. Hexazinone (17), Oxyfluorfen (0.037), Pendimethalin (0.0063), Trifluralin (0.0003) – compound-specific US-EPA Endangered Species Act risk-assessment documents, chronic fish NOEC/NOEL (most sensitive species, typically fathead minnow).

6. Imazapic, Isoxaflutole, Sulfentrazone, Tebuthiuron, Trifloxysulfuron (all 0.0001 mg L^{-1}) – generic EU parametric value for an individual pesticide in water intended for human consumption, Directive (EU) 2020/2184, Annex I, Part B (0.1 μg L^{-1}; unchanged from the original Council Directive 98/83/EC of 1998). This is not a compound-specific ecotoxicological endpoint, but a regulatory default applied uniformly across all individual pesticides in EU drinking water. Barreto et al. (2020) reported the same numeric value for two of these compounds (clomazone and sulfentrazone), sourced there to a generic placeholder for unknown C_{max}; a compound-specific EPA chronic-fish value was subsequently located for clomazone (tier 4 above), while sulfentrazone retains the generic EU value, arrived at independently here rather than carried over from that source.

Five conceptually distinct categories of C_{max} are therefore combined in this dataset: a Brazilian legal water-quality standard (tier 1), a value drawn from the foundational paper's own worked example (tier 2), US risk-screening benchmarks (tiers 3–4), and an EU human-health drinking-water default applied as a blanket substitute where no compound-specific figure could be located (tier 5). These differ in regulatory weight and derivation method; this heterogeneity, and its effect on the R ratio, is discussed explicitly in the main text (Sections 2.7, 3.3, and 4.3), which reports R both for the full 17-compound set and for the 12-compound subset restricted to tiers 1–4.

Herbicide	HRAC group	A_D (kg ha^{-1})	A_C (ha)	EC$_{50}$ algae (mg L^{-1})	EC$_{50}$ daphnids (mg L^{-1})	EC$_{50}$ fish (mg L^{-1})	$t_{1/2}$ (day)	K_{oc} (L kg^{-1})	α	AF
Ametryn	C1	2.23	1.88×10^6	0.0037	28.0	1.060	60	300	0.0110	3.2×10^{-18}
Amicarbazone	C1	1.00	3.36×10^4	0.084	0.252	13.0	54	37	0.0470	8.7×10^{-6}
Carfentrazone	E2	0.04	3.36×10^4	0.0127	9.8	0.0164	3	750	0.0001	~ 0
Clomazone	F4	1.00	1.52×10^6	3.5	5.2	19.0	24	300	0.0070	1.9×10^{-44}
Diuron	C1	1.83	1.00×10^6	0.0024	0.113	0.0618	90	480	0.0080	2.7×10^{-18}
Glyphosate	G	1.62	9.22×10^5	2.2	3.0	1.3	47	24000	0.0001	~ 0
Hexazinone	C1	0.29	8.51×10^5	0.0068	33.1	100.0	90	54	0.0450	2.6×10^{-4}
Imazapic	B	0.22	6.69×10^5	0.0523	100.0	98.7	90	1	0.1130	1.4×10^{-2}
Imazapyr	B	0.33	5.02×10^5	12.2	100.0	100.0	90	100	0.0300	7.9×10^{-6}
Isoxaflutole	F1	0.16	3.03×10^5	0.14	1.5	1.7	100	400	0.0100	3.6×10^{-14}
Metribuzin	C1	1.58	2.78×10^5	0.0081	4.18	42.0	40	60	0.0310	3.0×10^{-9}
Oxyfluorfen	E2	2.00	1.32×10^4	0.0003	0.08	0.17	35	5000	0.0010	~ 0
Pendimethalin	E	1.38	2.53×10^5	0.0054	0.28	138.0	90	5000	0.0010	~ 0
Sulfentrazone	E2	0.70	7.08×10^4	0.031	60.4	93.8	540	887	0.0060	7.1×10^{-6}
Tebuthiuron	C1	1.00	6.06×10^4	0.05	297.0	106.0	360	80	0.0440	7.7×10^{-2}
Trifloxysulfuron	B	0.04	5.31×10^4	0.0065	108.0	103.0	78	1	0.1100	7.2×10^{-3}
Trifluralin	E	0.80	3.32×10^4	0.339	0.56	0.0007	60	8000	0.0001	~ 0

C_{max} column (mg L^{-1}), by tier (see key above): Ametryn 0.0014^b; Amicarbazone 7.3^d; Carfentrazone 0.118^d; Clomazone 0.35^d; Diuron 0.0002^c; Glyphosate 0.065^a; Hexazinone 17^e; Imazapic 0.0001^f; Imazapyr 0.036^c; Isoxaflutole 0.0001^f; Metribuzin 0.001^c; Oxyfluorfen 0.037^e; Pendimethalin 0.0063^e; Sulfentrazone 0.0001^f; Tebuthiuron 0.0001^f; Trifloxysulfuron 0.0001^f; Trifluralin 0.0003^e.

[a] CONAMA Resolution n.º 357/2005, Art. 14–15 (freshwater Classes 1–2 standard); independently cross-checked against Barreto et al. (2020), Table 1, which reports the identical value.

[b] US-EPA Reregistration Eligibility Decision (RED) for ametryn, groundwater acceptable concentration limit, as cited directly within Paraíba et al. (2014) (Discussion section, H-model worked example).

[c] Barreto et al. (2020), Table 1 (joint citation: CONAMA and US-EPA Pesticide Reregistration Eligibility Decision database); reported here as given in that secondary source.

[d] US-EPA Office of Pesticide Programs, Aquatic Life Benchmarks for Pesticides (freshwater vertebrate, chronic value).

[e] US-EPA Endangered Species Act risk-assessment documents (chronic fish NOEC/NOEL, most sensitive species, typically fathead minnow): oxyfluorfen (rainbow trout, 37 μg L^{-1}), hexazinone (fathead minnow, 17,000 μg L^{-1}), trifluralin (fathead minnow, 0.3 μg L^{-1}), pendimethalin (fathead minnow, 6.3 μg L^{-1}).

[f] Generic EU individual-pesticide parametric value, Directive (EU) 2020/2184, Annex I, Part B (0.1 μg L^{-1} = 0.0001 mg L^{-1}), applied uniformly to imazapic, isoxaflutole, sulfentrazone, tebuthiuron and trifloxysulfuron in the absence of a compound-specific value. Not compound-specific; see the tier-heterogeneity note above.

References

Barreto, M.L.P., Netto, A.M., Silva, J.P.S. da, Amaral, A., Borges, E., França, E.J. de, Vale, R.L. (2020). Gray water footprint assessment for pesticide mixtures applied to a sugarcane crop in Brazil: a comparison between two models. *J. Clean. Prod.*, 276, 124254. https://doi.org/10.1016/j.jclepro.2020.124254

CONAMA (2005). Resolução n.º 357, de 17 de março de 2005. Conselho Nacional do Meio Ambiente, Brasil. Diário Oficial da União n.º 53, 18/03/2005, p. 58–63.

Directive (EU) 2020/2184 of the European Parliament and of the Council of 16 December 2020 on the quality of water intended for human consumption (recast). *Official Journal of the European Union*, L 435, 23.12.2020, p. 1–62, Annex I, Part B. https://eur-lex.europa.eu/eli/dir/2020/2184/oj/eng

Hornsby, A.G., Don Wauchope, R., Herner, A.E. (1996). *Pesticide properties in the environment.* Springer-Verlag, New York.

US-EPA, Office of Pesticide Programs (n.d.). Aquatic Life Benchmarks and Ecological Risk Assessments for Registered Pesticides. https://www.epa.gov/pesticide-science-and-assessing-pesticide-risks/aquatic-life-benchmarks-and-ecological-risk

US-EPA (2001). Oxyfluorfen: Analysis of Risks to Endangered and Threatened Species.

US-EPA (2004). Hexazinone: Analysis of Risks to Endangered and Threatened Salmon and Steelhead.

US-EPA (2004). Trifluralin: Analysis of Risks to Endangered and Threatened Pacific Salmon and Steelhead.

US-EPA (2004). Pendimethalin: Analysis of Risks to Endangered and Threatened Salmon and Steelhead.

Paraíba, L.C., Pazianotto, R.A.A., Luiz, A.J.B., Maia, A.H.N., Jonsson, C.M. (2014). A mathematical model to estimate the volume of grey water of pesticide mixtures. *Span. J. Agric. Res.*, 12(2), 509–518. https://doi.org/10.5424/sjar/2014122-4059
